# The Appeal of Ethnobotanical Folklore Records: Medicinal Plant Use in Setomaa, Räpina and Vastseliina Parishes, Estonia (1888–1996)

**DOI:** 10.3390/plants11202698

**Published:** 2022-10-13

**Authors:** Renata Sõukand, Raivo Kalle

**Affiliations:** 1 Department of Environmental Sciences, Informatics and Statistics, Ca’ Foscari University of Venice, Via Torino 155, Mestre, 30172 Venice, Italy; 2 University of Gastronomic Sciences, Piazza Vittorio Emanuele II 9, 12042 Pollenzo, Italy; 3 Kuldvillane OÜ, Umbusi, Põltsamaa v, 48026 Jõgeva, Estonia

**Keywords:** historical ethnobotany, folklore collections, biocultural diversity, Estonian history, folk medicine, medicinal plants

## Abstract

The historical use of medicinal plants is of special interest because the use of plants for healing is a rapidly changing, highly culture-specific and often need-specific practice, which also depends on the availability of resources and knowledge. To set an example of folkloristic data analysis in ethnobotany, we analyzed texts from the database, HERBA, identifying as many plants and diseases as possible. The research was limited to the Seto, Räpina and Vastseliina parishes in Estonia. The use of 119 taxa belonging to 48 families was identified, of which nine were identified at the genus level, four ethnotaxa were identified as two possible botanical taxa and fifteen ethnotaxa were unidentifiable. The most frequently mentioned taxa were *Pinus sylvestris*, *Matricaria discoidea* and *Valeriana officinalis*. High plant name diversity as well as high heterogeneity in the plants used were observed, especially in earlier records. The use of local wild taxa growing outside the sphere of everyday human activities, which was abandoned during Soviet occupation, signals an earlier, pre-existing rich tradition of plant use and a deep relationship with nature. Working with archival data requires knowledge of historical contexts and the acceptance of the possibility of not finding all the answers.

## 1. Introduction

Historical ethnobotanical data in folklore archives are often perceived romantically as reservoirs of indigenous plant use, even in the European context. In addition, having locality-based historical data for comparison with currently existing plant uses can reveal tendencies in the evolution of plant use. The historical use of medicinal plants is of special interest because the use of plants for healing is a rapidly changing, highly culture-specific [1] and often need-specific practice, which also depends on the availability of resources and knowledge (e.g., advances in public health). Yet, studies based on historical ethnobotany are largely underrepresented in current scholarship [2]. The food-related historical ethnobotany of Northern Europe is somewhat better researched, represented by analyses of the collections of the Polish botanist, Józef Rostafiński (1850–1928) [3,4], given that they contain herbarium specimens. However, examples from the medicinal plant perspective are rather rare: there is an analysis of the first citizen science-based identification in Estonia, of which, however, only the interpretations of the contributions by the collector have survived [5]. An earlier medicinal plant study, by the pastor Johann Heinrich Rosenplänter (1782–1846) of the Pärnu parish [6], dates back to the beginning of the 19th century and can be considered as one of the first ethnobotanical studies in the Baltic countries [6]; however, this too was based on already processed information and not folklore collections.

The difficulty in identifying plants is the primary reason why the historical ethnobotanical records, preserved among other folklore and/or local history collections in archives, are mainly neglected and excluded from current scientific discussion. The highest risk in dealing with such data lies in the absence of attached herbarium specimens, which, in a situation of ambiguity with respect to the local plant name, creates a lot of doubt regarding the reliability of the connection between the emic (local plant name) and etic (scientific name of the taxon). Such material is also often collected *en passant*, along with other folklore-related records, such as folk songs and mythology. Such records are documented only few at a time and therefore do not seem very informative.

There are quite a few databases containing ethnobotanical data [7], but only some of them have thus far focused exclusively on archival data. Databases such as HERBA in Estonia [8] and Dúchas in Ireland [9] enable the combination of data from several folklore collections. Notably, throughout the 19th and 20th centuries in Northern and Central Europe, folklore collections had specific questionnaires or campaigns which were designed keeping ethnobotanical principles in mind (although not always acknowledged during the time of collection) or, even more, the Latin names were added by botanists, so the plants have already been identified. Yet even in such cases, great attention must be paid to the details and mass analysis is rarely possible. In addition to datasets, attention needs to be given to possible misinterpretations and deliberate plagiarism, especially if the collection campaign had a competitive nature or the report was composed by schoolchildren, as is the case for several collections deriving from the 20th century, for example in Ireland [10] and Estonia [11].

The robust transformation of qualitative data in archives into quantitative data has become increasingly common, especially among researchers with a pharmaceutical background. Since today’s European ethnopharmacology is largely influenced by the literature and ethnobotanical fieldwork rarely finds new uses for medicinal plants, more and more researchers have started to look at the data stored in archives. This primarily concerns unexplored archives in former socialist countries with large folklore collections; e.g., in Lithuania, the folk plant use data stored in archives have not yet been thoroughly studied [12]. However, Estonian (e.g., [13]) and Latvian [14,15] pharmacists have already published articles based on archival data without a prior thorough critical analysis of the sources. When processing archival data, there should be a strong emphasis on metadata, which pharmacologists, however, do not know how to process. It is very important to evaluate the time at which the data was collected, who collected it and with what methods, what the motivation for the collection was, etc. Such an analysis also needs to explain how plant species and diseases were identified. It is also important to describe in the Section 4, how the qualitative data, which could have been collected at different times and with different methods, were quantified and how the categorization took place. However, researchers with a pharmaceutical background do not provide such information (e.g., [13,14,15]). There are also no references in these above-mentioned articles revealing in which archive collections the original data are located. It can be said that there are major errors in such articles based on already published Estonian data, which gives reason to believe that there are also inaccuracies in the data for other countries.

Borderlands between cultures have been of increasing interest to ethnographers (e.g., [16,17]) and ethnobotanists (e.g., [18]) for understanding the interaction of different ethnic groups and their knowledge circulation. Therefore, we focused our case study on a limited geographic area (three historical parishes) in the Estonian and Russian borderland. In order to illustrate how to deal with the difficulties of plant identification and data interpretation, we examined seven folklore collections from the Estonian Folklore Archive, the oldest reports of which date from 1888 and the latest, from 1996. Ethnobotanical fieldwork also recently took place in this region [19] and, therefore, we used part of these data in comparison with that work.

### 1.1. Seto (Setomaa), Räpina and Vastseliina Parishes

**Figure 1:**
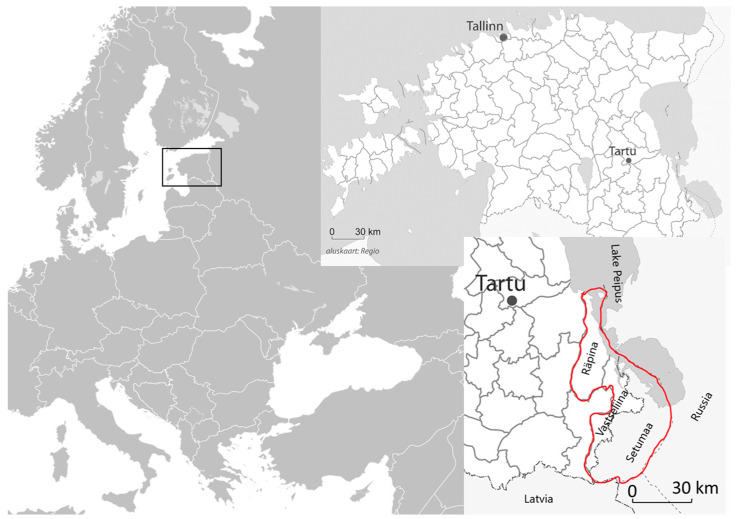
Map of the region.

Since Estonian folkloristics and ethnography are based on historical parish boundaries, we also followed this principle in our work. The reason for this is because historical parish borders remained more or less unchanged in Estonia until the end of the Estonian War of Independence. The two Estonian parishes (Räpina and Vastseliina) closest to the Russian border and the closest Russian border area (Seto) were chosen as the research area (Figure 1). After the Estonian War of Independence (1918–1920), the Setomaa area was incorporated into the Republic of Estonia, and following World War II, most of this area was incorporated into the Russian SFSR. The historical parish boundaries changed due to numerous reforms and today lie within the territories of several municipalities. However, the research area is sparsely populated; for example, in 2020, the Räpina municipality had a little over 6100 people, the Setomaa municipality (the Estonian part of the Seto area), a little over 3100 and the Võru municipality (most of Vastseliina parish belongs to it today), about 10,600 [19,20].

Since these are border regions, Russians and Finno-Ugric peoples have lived there, side by side, in different villages. Räpina and Vastseliina are predominantly Lutheran areas, and Setomaa is predominantly Russian Orthodox, but paganism is also widespread. In the parishes of Räpina and Vastseliina, large households (manors) were common historically, as elsewhere in Estonia, but only small farms were common in Setomaa. Since Setomaa was located on the outskirts of Russia, it was a very poor area where farmers had little land and were mainly engaged in vegetable growing, handcrafting, trading and fishing. Nature in the region is greatly influenced by Lake Peipus, as well as the Haanja Upland (highest elevation: 318 m a.s.l.), with its hummocky landscape. Coniferous forests and, in wetter areas, swampy deciduous forests predominate. Agriculture, forestry and fishing are the main activities in the region today [19,20].

#### The Organization of Medicinal Support in the Region

At the end of the 19th century, there was still a large number of folk doctors or witches in Võru County. They were feared, but people went to them for help in times of illness [21]. By the beginning of the 20th century, however, in some regions of Võrumaa, folk doctors nearly disappeared. The reasons for this were the wider availability of school education, the explanatory work of the Christian Church and the greater presence of medical doctors in rural areas [22]. In addition to going to folk doctors, people in Võrumaa and Setomaa visited various healing stones, springs and trees that were found throughout the region. The most important ones, visited by people from all over the area, included Miikse Jaanikivi and Silmaallika, the Võhandu River (which is called Pühajõgi, or “holy river”, near the town of Võru), and the sacred oak of Pechory. On the Republic of Estonia’s side of the border, former “natural spas” have now been placed under nature or heritage protection. One of the reasons for such a popularity of natural healing objects may be the fact that in the 19th century, there were no doctors in the rural areas of the Livonian governorate (to which Võrumaa belonged), as they were located only in the towns [23], e.g., Võru and the neighboring governorate of Pechory. These towns also had rural hospitals and pharmacies, including in Võru (opened in 1827 and 1785, respectively) and Pechori (c. 1890s and 1865, respectively). Pharmacies and general stores were established in the rural areas and larger settlements of the Livonian governorate in the early 20th century, e.g., in Rõuge (opened in 1896), Räpina (opened in 1861) and Leevi (opened in 1910). The opening of pharmacies particularly increased after the Estonian War of Independence: Lepassaare (1927), Misso (1925), Osula (1924), Irboska and Värska in 1923 [24]. The Soviet Union unified the medical system and the official health care was free; however, it was not always efficient, so people also looked for alternatives. The use of plants was promoted by the state medical system as well as by the procurement of medicinal plants through pharmacies, and as a result, the use of plants was very popular throughout this time.

### 1.2. The Aim of the Work

This article has two main objectives:

(1) to analyze the historical material and compare the two regions; and
(2) to provide an example of how to treat archival data in ethnobotanical research, in such a way that it is fully useable in modern scientific research and comparable with currently obtained data.

To this end, we:

(a) reviewed folklore ethnobotanical texts from the HERBA database [8], and identified the various plants and diseases whenever possible; and
(b) limited our research to the whole of Setomaa and only the two parishes of Võru (Räpina and Vastseliina) in order to have a relatively comparable number of texts.

From the start, we anticipated that we would not be able to identify all the plants and diseases described in the texts. However, the remaining dataset is sufficient to contribute to the analysis of the influence of the Soviet period on the local ecological knowledge of the region.

## 2. Results

### 2.1. Cleaning the Data

All the texts initially selected from HERBA were carefully read and evaluated for their suitability for the analysis. There were specific records that were not incorporated into the analysis, even though present in HERBA, because:

- the text clearly referred to a pharmacy drug or a processed product purchased from a travelling merchant;
- no clear reference to the plant was given (e.g., make the broom from nine leafy trees);
- students supplying the records clearly misinterpreted the information and/or clearly confused the plants;
- there was no reference to the plant’s application (stating that it was just medicine);
- poisonous aspects of a plant were highlighted with no medicinal application; and
- when students used both the Latin name and local name of the plant deriving from literary sources and a clear literature influence could be detected.

If the use was copied and shared by more than one student, only one text was retained for analysis. There were many such cases of students “working together”. For example, in 1929, nine students from a Värska school referred to bathing with *Trifolium* to treat rheumatism, writing the sentence identically. It is noteworthy that a similar use was also described (with more details and different wording) in a text from 1937 and the same taxon was claimed to be used against typhus (which is a little suspicious, although we cannot completely rule it out).

Greatly overlapping texts also sometimes originated from two different generations and some of them could not be attributed to tradition. For example, a text from 1985, referring to the notes left by a woman who died in 1984 at age 92, is almost identical to the one written by a male student from a Pugola elementary school in 1937. The text refers to the treatment of cancer with a long list of plants that are boiled together. We were able to trace that the texts were copied from a newspaper published in September 1936 that discussed Tallinn’s herb sellers and how they taught people to use medicinal plants [25].

One exception can be highlighted—*Linum ussitatisumum*—which was often used in the form of a linen cloth (not a living or dried plant), as it was stressed that the used fabric had to be made from linen, even though it was a very common fabric. Such texts were included.

### 2.2. The Identification of Plants

As is very common for local plant names, even in a small community, one name may refer to different species; in this situation, the description of the disease’s origin and the habitat of the taxon were consulted. In the example of *kärnahain* (“scab herb”), two different *Rumex* taxa were associated with the texts. One was *Rumex crispus*, a widespread name that had already been identified in a text collected by pastor Jakob Hurt in 1903. The second text had no identification, but a description of the plant (“being dug out of the ground”) was provided, which clearly refers to one growing on dry land (hence, *R. crispus*). However, the third text very likely refers to *Rumex hydrolapathum* Huds., which grows in wetlands, as the text describes a disease that derives from water.

The later the text, the more chances there were that the names had already been unified. A good example is provided by *verihein* (“blood herb”), a name that in 19th-century Setomaa could be attributed to *Argentina anserina* or *Achillea millefolium*. Here, the description of the plant is of crucial importance. For example, in a text from 1889, *verihain* is described as having leaves as those of ash (*Fraxinus excelsior*) and little yellow flowers—this refers to *Argentina anserina*. The other examples were from later times (starting from 1937), where only the use was provided (mainly cough and staunching blood). By that time, plant names were more unified and the same name could now be associated with *Achillea millefolium*, for which *verihain* was a widespread name throughout Estonia and such a use, widely known. However, one of the earliest texts (collected by Jakob Hurt in 1903) identified *Achillea millefolium* as *verihain*, wherein the use against lung disease was described.

*Arnika* is a local plant name that was very difficult to identify. *Arnica montana* L. (to which the name refers) does not grow in Estonia, however, according to botanist Gustav Vilbaste’s book of plant names, two taxa (*Scorzoneroides autumnalis* or *Solidago virgaurea*) are most often identified with this name, although there is always the possibility for other taxa being used (see [26] for more details).

Fifteen local names (ethnotaxa) remained unidentified for various reasons. One example is *pinipussuhain* (“plant smelling like dog fart”), a name that could be related to several plants in the region and for which there was no description of the plant or any other potentially helpful details. It was used to treat lung diseases and therefore most likely refers to *Hyoscyamus niger*; however, it could also refer to *Ballota nigra* and *Ranunculus acris* (name uses unique to our region), or *Mentha arvensis* (a name widespread throughout Estonia), and so it remained unidentified. Another quite representative example is *luuvaluheinad* (“bone pain herbs”), whose specific form in the Viljandi parish referred to *Persicaria amphibia* (L.) Delarbre, while in Räpina, from where this text derived, a similar name (*luuvalurohi*) was recorded for *Polygonatum odoratum* (Mill.) Druce. The meaning of both names is the same, namely medicine for bone pain, and throughout Estonia, ten taxa had similar names. Therefore, the name is too ambiguous to be identified without further explanation or description. *Valge lill* refers to the color of the flowers (white) and without a description, the actual plant used is not identifiable and could represent various taxa even for the same use category.

The cases in which there was more than one plant name provided, helped to facilitate plant identification. For example, the local name *jumalakäpp*, refers to orchids from several different families (i.e., *Orchis* sp., *Platanthera bifolia* and *Dactylorhiza maculata*), yet in Setomaa, the name *juudakäpp*, refers to *Dactylorhiza maculata*, and therefore identification was possible.

One rather misleading plant name in the region is *takja*. In general (all across Estonia), this name refers to *Arctium tomentosum*, yet when it is used against cough, it refers to *Cetraria islandica* (L.) Ach., which is more often called *palotakja*.

### 2.3. The Identification of Etic Disease Categories

For establishing clear and countable use records (UR), specific ad hoc rules were followed as outlined below.

Sometimes, there were cases in which the use of one or several plants was potentially described by several symptoms referring to one disease. In the majority of cases where the use was described with two or more disease names or symptoms belonging to the same etic disease group, the text was treated as one UR. For example, foot and back pain remained symptoms of one disease belonging to the musculoskeletal etic disease group. When the disease was provided with symptoms, such as wounds or swelling and pain, it was recorded as a wound (and thus the dermatological disease category). Another example is when the plant was described as promoting sweating and against cold; since both the disease (cold) and symptom (sweating) are related to the general disease category, it was recorded as one UR.

From these rules, a few exceptions were made where the emic symptoms or diseases described together belonged to different etic disease categories, for example, in the cases of cough and lung diseases or tuberculosis. The former (cough) clearly belongs to the respiratory disease group. However, *kopsuhaigus* (“lung disease”) was often a popular name for tuberculosis as well as some other infectious lung diseases, and although proper diagnostics for tuberculosis in the region were restricted, a long-lasting cough and other symptoms of tuberculosis or severe infections affecting the lungs were well differentiated from the “ordinary” cough, and we therefore attributed them to infectious diseases. We also had one example where *tiisikus* (“tuberculosis”) and *tüüfus* (“typhus”) were treated with the same plant—both are infectious diseases, yet very different in nature and therefore counted as two different UR. Tooth diseases and stomach diseases were also accounted for separately. Another example is that of stomach disease, liver disease and hemorrhoids, as all three belong to the gastrointestinal etic disease category, yet they were considered separately as they refer to different organs. A student from Räpina parish reported the use of a strong tea made from *arnika* to treat internal pain (*seest valu*), which was attributed to the culture-bound disease category and diarrhea (digestive category).

Diseases or symptoms with deep mythological connotations, such as *kaetus* (evil eye), *halltõbi* (“grey disease”) (malaria), *seest haigus* (“internal disease”) and *venitus* (“internal tension”) (muscle pain, usually due to hard work), *pistja* (“stabbing pain”) and *vaivaja* (nightmare), which were not univocally interpretable, were classified as belonging to the culture-bound disease category, which does not exist in the International Classification of Primary Care, 2nd edition [27]. There was a difficult decision to make in the case of *jooksva* (“runner”), which refers to pain changing its location in the body. The selection of the plants used to treat it has also historically been related to some perceived properties of the disease (such as an origin from a wet place or creeping along the ground) [28]. However, as the disease is closely related to the ailment currently known as rheumatism (and this name was also often mentioned), it was attributed to the musculoskeletal disease category (Table 1).

### 2.4. A General Overview of Plant Uses

After cleaning the data, 1072 UR were retained for the analysis (Table 2).

The data were provided by 47 correspondents, 13 of whom provided just one or two UR. Among the correspondents were several folklorists who visited two or all three of the parishes, including the founders of the Estonian Folklore collections, namely Jakob Hurt, who collected 27 UR from Setomaa, and pastor Matthias Johann Eisen (five UR) from Räpina. The most productive collectors were schoolteachers, who collected the work of numerous students for several years following the request of Gustav Vilbaste; J. Haring, a Värska primary school teacher in Setomaa, sent thirty-five student responses (438 UR) from the area and one student response (13 UR) from Räpina parish. There was one other productive teacher, M. Kaasikmäe from the Setomaa Košelki primary school, who sent 11 student responses in total (70 UR). Anna Vitsust, a Räpina Gymnasium teacher, also sent twenty-one student responses (259 UR) from Räpina, and Kotlep Pärg, a Pugola elementary school teacher in Vastseliina, sent seven student responses (163 UR). There were two more responses provided by two students which we excluded from the analysis. One of them had only listed 57 medicinal plants without any specification about diseases, and the other student stood out for having recipes that were too detailed, all of which were copied from the above-mentioned newspaper article [25]. In addition, Gustav Vilbaste himself (as he was a teacher in the city of Tartu) collected the response of a student from Räpina who came to study at a Tartu school (20 UR). Of the other correspondents, important information was provided by volunteers, Daniel Lepson (farmer; 29 UR) and Maria Linna (agricultural worker; 27 UR), who collected village folklore by interviewing the local inhabitants.

The use of 120 taxa belonging to 48 families was identified, of which nine were identified at the genus level and four ethnotaxa were identified as two possible botanical taxa (Table 3). In addition, 15 ethnotaxa were unidentifiable and therefore they were left out of the formal analysis; however, their uses are presented in Table 3. The most represented families were Asteraceae (sixteen taxa + two potential taxa), Rosaceae (thirteen taxa) and Ericaceae (eight taxa). The most frequently mentioned taxa were *Pinus sylvestris* (57 UR), *Matricaria discoidea* (51 UR), *Valeriana officinalis* (50 UR), *Achillea millefolium* (42 UR), *Juniperus communis* (39 UR) and *Tilia cordata* (39 UR). Thirty-four taxa had only one UR, while thirty-five taxa had ten or more UR.

Of all the used taxa, 23 were cultivated, most of which were garden fruits, vegetables and crops. Only four plants can be said to have been cultivated as medicinal plants: *Mentha* spp. and *Matricaria chamomilla* in the garden, and *Aloe arborescens* and *Capsicum annuum* in flowerpots indoors. Most of the natural species on the list were common plants in Estonia. According to today’s understanding, only four plants are near threatened and *Gentiana* spp. is in the endangered category. The list also includes three herbs that do not grow in Estonia, which were bought in a shop.

The most represented etic disease categories were general, digestive and skin. The proportion of culture-bound diseases was also relatively high. The most often mentioned emic disease categories were cough (129 UR), stomachache (64 UR), tuberculosis (62 UR), cold (57 UR) and stomach disease (52 UR).

**Figure 2:**
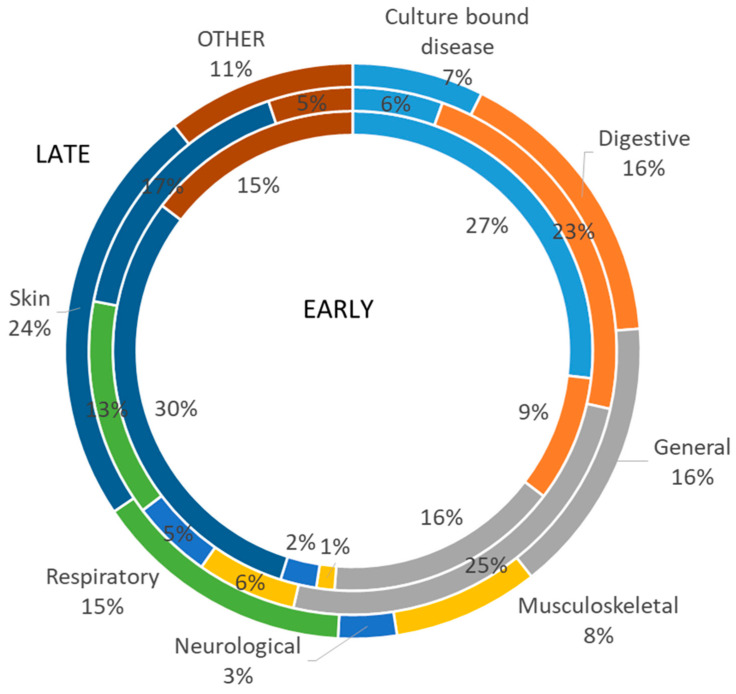
A proportional division of the general disease categories in early, middle and late folklore collections.

The proportional division of disease categories between the different times of collection illustrates the change in the importance of some of the categories throughout the century (Figure 2).

### 2.5. A Cross-Cultural and Diachronic Comparison

The cross-cultural comparison of the whole dataset shows high heterogeneity. Of the 119 taxa, 90 were recorded in the two Võro parishes, while 84, in Seto parish. Overall, 55 taxa overlapped (JI = 0.46), while 35 taxa overlapped for those recorded with three or more UR (JI = 0.54). Seto parish showed slightly greater consistency in the use of fewer taxa (52 out of 84 had three or more UR), while the Võro parishes exhibited more diversity (almost half of the used taxa (43 out of 90) had less than three UR). A few taxa, represented by three or more UR, were characteristic of one group; *Taraxacum officinale*, *Menyanthes trifoliata*, *Hordeum vulgare*, *Daphne mezereum*, *Angelica sylvestris* and *Dryopteris filix-mas* were used exclusively in the Võro parishes, while *Malus domestica*, *Beta vulgaris*, *Prunus ceraseus*, *Brassica oleraceae*, *Drosera rotundifolia*, *Persicaria amphibia*, *Rumex*, *Gentiana pneumonanthe* and *Urtica urens* were reported exclusively in Seto parish.

However, we need to take into consideration the fact that the folkloristic data was collected very unevenly. If we look at the data collected within the early period (before 1904), we can observe that many disease categories are represented only in one parish, which is illogical.

**Figure 3:**
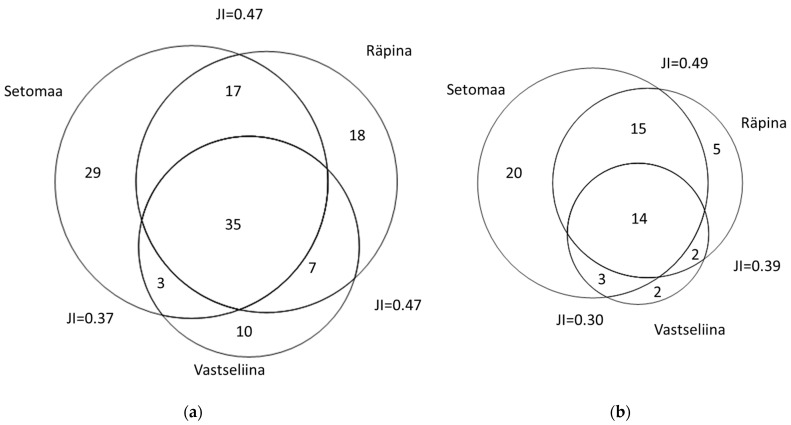
A comparison of (**a**) all plants and (**b**) those used in three or more UR in the three studied parishes. JI—Jaccard Index.

The comparison between all three parishes increases the diversity; however, we need to consider that the low number of UR from Vastseliina is most likely due to the lack of data, not the lack of actual uses (Figure 3).

**Figure 4:**
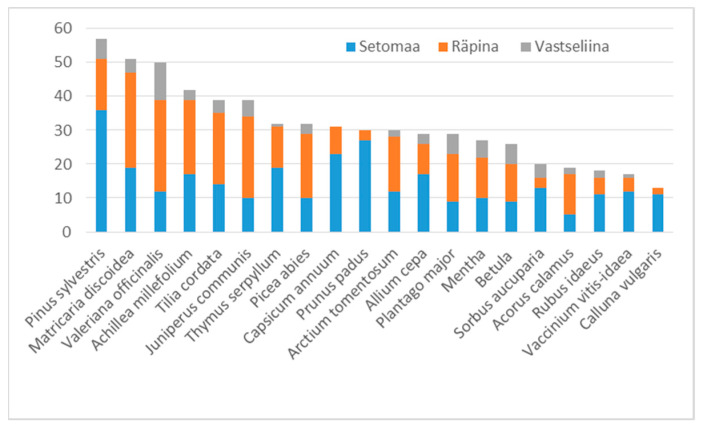
The 20 most used taxa and their UR distribution in the parishes.

The 20 most used plants based on the number of UR, with a few exceptions (*Prunus padus*, *Capsicum annuum* and *Calluna vulgaris* were not mentioned in the Vastseliina parish), were present in all the parishes, but the proportion of use is not even (Figure 4).

## 3. Discussion

The Jaccard Indexes (JI) obtained in the cross-parish comparison are remarkably lower than those from the recently collected data [19]. It should be kept in mind that these only represent identified taxa, while unidentified ethnotaxa were not taken into account in the calculation of the JI. A high number of cultivated species was present exclusively in the Seto material, which is noteworthy as the use of cultivated species for medication is more characteristic of Slavic communities [29], indicating that the Setos had closer contact with neighboring Russians (see also [30]).

In the texts, there was a high proportion of diseases related to culture (over 6%), yet proportionally, this was higher in the early period and almost absent in the late (occupation) period. This is consistent with the tendency seen in the recently obtained data where culture-bound diseases were completely absent from the disease list [19]. The prevalence of the digestive disease category in the dataset from the 1930s may also be due to the fact that the data was collected by students, which limited to some extent the diseases covered by the data. The absence of respiratory diseases among the early data is quite indicative of historical data, guided by the understanding that minor diseases such as a runny nose or simple cough were not considered worth mentioning in peasant society and were often not even treated.

The presence of 15 unidentifiable folk taxa does not diminish the data obtained; instead, it shows the high diversity of plant names, especially in the early dataset, and the potential presence of ad hoc names. The high diversity of local plant names (mainly wild taxa reported by one person or cultivated ones that have only a single local name) demonstrates the historical diversity of plant names within the very limited geographic area. The presence of local names referring to a disease (primarily from the early and middle datasets) presents an important aspect of Estonian folk medicine, which was previously highlighted by Jakob Hurt in his identifications in 1888 [31], yet has been seldom addressed in the international literature thus far.

The use of local wild taxa growing outside the sphere of everyday human activities (such as *Eriophorum vaginatum*, *Menyanthes trifoliata*, *Dactylorhiza maculata*, *Daphne mezereum*, *Paris quadrifolia*, *Chimaphila umbellate*, *Andromeda polifolia and Persicaria amphibia*), which was abandoned during Soviet occupation, signals an earlier, pre-existing rich tradition of plant use and a deep relationship with nature through seasonal activities and general interaction with the surroundings. There were several reasons behind this, including the introduction of standardized medicinal plants during the Soviet era, a change in rural lifestyle and the replacement of extensive agriculture (small farm systems) with intensive agriculture (collective farm systems), causing drastic changes in rural life.

Although from today’s nature conservation point of view, the majority of used local medicinal plants have a low extinction risk (Least Concern status), their general conservation status does not reflect their future sustainability. As we have shown in a recent study, even common plants in the immediate vicinity of humans may be disappearing because of changes in the management of semi-wild areas [32].

Abandoned specific cultivated species (such as *Secale cereale*, *Linum usitatissimum*, *Brassica rapa* and *Papaver somniferum*) can help to track changes in cultivation habits. For example, *Papaver somniferum* disappeared from use because it was proclaimed to be a narcotic and its cultivation in gardens was prohibited, while *Linum usitatissiumum* processing and linen cloth lost its importance with the appearance of new and more affordable textile products.

While working with pre-systematized archival datasets (Table S1), there are some important aspects to consider:

(1) Not all pre-systematized data may be suitable for the research purpose, and therefore some of the data may need to be removed due to not having sufficient information (as we did with missing information on a specific identified use).
(2) One needs to be critical of the data, especially if the collection has some competitive undertones and there is a possibility of group work or risk that the actual data was beautified.
(3) It is better to under-identify the data than over-identify it; working with historical data, especially folklore material, it is inevitable that some taxa remain unidentified.
(4) It is important to involve specialists in the local flora also having a background in historical biogeography.
(5) Working with such a dataset also requires knowledge of the purchasable material in the region.
(6) The historical epidemiology of the region also needs to be studied prior to disease identification.

Despite our efforts and previous experience in plant and disease identification in historical data, we were not able to precisely identify approximately 20% of the taxa whose uses were provided in the texts. While identification at only the genus level is inevitable when dealing with such taxa that are also not differentiated by the people themselves, the potential misidentification at the genus level (when the local name can be attributed to two or more species) can be problematic from an ethnopharmacological point of view. Although the unidentified ethnotaxa may seem like a complete loss from an ethnopharmacological perspective, they may bear important information from a cultural and ethnographic perspective, and signal the richness of the used flora.

Therefore, it should be kept in mind that there is no one-size-fits-all solution for data preparation and analysis, and therefore the utmost care needs to be taken in data interpretation in order to avoid mistakes. Working with archival data requires good preparation and the study of the historical context of the data under investigation, as well as open-mindedness and the ability to accept the fact that not all questions will be answered.

## 4. Materials and Methods

The basis of the analyzed texts was the HERBA database, a relational database designed by the authors [8], in which herbal folk medicine texts are searchable by local plant name and emic disease category. The HERBA database is based on numerous folklore collections housed in the Estonian Folklore Archives of the Estonian Literary Museum; most of the information has been sent in by various correspondents or collected through folklore expeditions. To construct HERBA, the texts were first identified from their original sources (Table 4) by using the registration of the collection (if present) and by carefully reading the texts within. Vilbaste’s collection was worked through entirely. Plant use-related texts were transcribed (for which technical assistants were employed), checked with the original and then entered into the HERBA database by the authors. Local plant names were correlated to the botanical taxa through an additional dataset composed of different sources, mainly relying on the book of Estonian folk plant names by Gustav Vilbaste [33], in which he compiled all existing information on local plant names, and on HERBA [8] itself. We also identified folk diseases based on these two sources.

The resulting data were extracted from the database in an Excel format and the text segments (the complete narrative units corresponding to one or more plants used for one or more emic diseases) related to the selected regions were separated out for the analysis. Seven of the folklore collections housed in the Estonian Folklore Archives of the Estonian Literary Museum contained data on the medicinal use of plants for the selected regions (Table 4). As the number of resulting texts for the three parishes was uneven, some of the comparisons were made after combining texts from Vastseliina and Räpina, in order to maintain a balance and also because both parishes were historically part of Old Võromaa.

The Excel spreadsheet was further processed and divided into conditional use records (UR), referring to the plant taxon used for treating the specific health condition reported by one correspondent. Emic disease categories provided in the texts were interpreted according to current knowledge and correlated to the medicinal categories of the International Classification of Primary Care, 2nd edition (ICPC-2) [27] (hereafter etic disease categories). As there were some emic diseases described that were not univocally correlatable to the ICPC-2 classifications, we created an additional category of culture-bound diseases (CBD). Within the CBD category were included such emic diseases as the evil eye or nightmare, as well as diseases represented by specific, culturally significant disease names, such as *seest haigus*, which could correspond to either the digestive, musculoskeletal or general disease category, yet were well positioned in the culture as phenomena.

For comparative purposes, the resulting dataset was divided into three temporal categories:

1. Early: 1888–1904 (80 UR);
2. Middle: 1928–1942 (871 UR); and
3. Late: 1949–1996 (121 UR).

The Latin plant names provided in HERBA (which was formed on the basis of the Estonian Plant Name database (https://taimenimed.ut.ee/ accessed on 10 July 2022) and our resulting identifications followed those listed in the Plants of the World Online (POWO) database [34] and the European Flora [35]; family assignments followed the Angiosperm Phylogeny Group (APG) IV [36]. The correlation between the emic plant name and plant taxon was carefully checked as described above, and a number of taxa remained unidentified, e.g., at the level of ethnotaxa.

### Data Comparison

The Jaccard Similarity Indices (JI) followed the methodology of González-Tejero et al. [37]: JI = (C/(A + B − C)), where A represents the number of taxa in sample A, B is the number of taxa in sample B, and C is the number of taxa common to A and B.

The proportional Venn diagrams were created using the PAST Toolkit Venn diagram plotter software program (https://omics.pnl.gov/software/venn-diagram-plotter (accessed on 10 July 2022)). The figures were visualized using RAW Graphs (RAW) [38].

## 5. Conclusions

Our results show a high diversity of historical medicinal plant use in three little parishes in Estonia and document the abandonment of numerous taxa growing outside the sphere of everyday human activities, which signal a deep knowledge of the wild. We also found that traditional medicinal plant foraging did not endanger local plant communities, as the majority of used plants were either very common and not endangered or cultivated, and therefore conservation should be more concerned with the reasons for the disappearance of common plants as the result of the decrease of human activities in rural areas.

We can conclude that archival data has great potential for revealing comparative data for current field studies and for understanding the historical context of medicinal plant use. However, when working with archives, the research methodology has to be carefully selected and adapted to the specific collection, while the results may not be exhaustive from the point of view of the identification of plants and diseases.

## Figures and Tables

**Table 1:** The relationship between emic and etic disease categories and the number of UR. **Bold** text corresponds to etic disease categories and their summary count. Etic/emic categories: No. of UR.

Blood: 8	Urological: 20	Respiratory: 134
blood cleaning: 5	blood peeing: 3	cough: 129
blood pressure: 1	kidney and bladder disease: 10	difficulties in breathing: 1
poor blood: 2	kidney disease: 5	nose bleeding: 2
	oedema: 1	sore throat: 2
	urinary retention: 1	
**Pregnancy, etc.: 3**	**Female genital: 11**	**Cardiovascular: 15**
giving birth: 3	problems with menstruation: 4	heart diseases: 15
	women diseases: 7	
**Culture-bound disease: 79**	**Musculoskeletal: 64**	**Neurological: 49**
*kaetus* (evil eye): 3	backache: 4	calming: 6
*halltõbi*: 4	bone pain: 3	epilepsy: 1
internal disease: 38	foot diseases: 4	headache: 13
*kidi* (tendovaginitis or bursitis in the wrist): 11	joint disease: 1	nerve disease: 8
*vaivaja, vaivajatõbi* (nightmare or hernia): 3	joint dislocation: 4	paralysis: 2
*pistja* (stixis, pleuritis, etc.): 6	*jooksva*/rheumatic: 48	seizures: 12
*riis* (umbilical hernia in children): 2		sleep disorder: 7
*tiir* (itchy soles): 1		
*tsirgutõbi* (a disease in young children): 1		
*ussiviga* (chorea in young children): 4		
**Skin: 200**	**Digestive: 227**	**General: 250**
abscess: 9	appendicitis: 1	against several diseases: 15
bee stings: 1	bile disease: 1	cold: 57
bleeding: 19	constipation: 5	cholera: 3
boil: 26	diarrhea: 12	disinfection: 6
burned wound: 13	digestion problems: 10	fever: 10
skin cancer: 1	gastric disease: 13	for sweating: 5
cracked lips: 4	gastric ulcers: 2	freezing: 6
cut wound: 2	hemorrhoids: 1	good for health: 4
dandruff: 4	heartburn: 2	inflammation: 2
eruption: 4	indigestion: 3	loss of appetite: 9
erysipelas: 22	jaundice: 8	lung disease: 34
for beauty: 4	liver disease: 6	pain: 3
fresh wound: 3	mouth diseases: 1	prophylactics: 4
hair loss: 1	nausea: 2	rabies: 1
inflammation: 7	stomach disease: 53	stroke: 2
itching: 1	stomach worms: 14	throat disease: 21
local pain: 2	stomachache: 64	tiredness: 1
lump on skin: 6	tapeworm: 4	tuberculosis: 62
pimples: 6	tooth diseases: 7	typhus: 2
*roos* (erysipelas): 2	toothache: 18	whooping cough: 2
rotten wound: 10	vomiting: 1	
scabies: 3		
scabs: 3		
skin disinfection: 2		
skin diseases: 1		
snake bite: 5		
splinter: 1		
sunburn: 1		
warts: 8		
wound: 28		
**Ear diseases: 2**	**Eye diseases: 10**	

**Table 2:** The use records (UR) remaining for analysis after the cleaning of the data and identification of the plants.

Collection/Parish	Räpina	Setomaa	Vastseliina	Sum
E	5	1	2	**8**
ERA	27	45	1	**73**
ERM			3	**3**
H	22	25	28	**75**
KKI		6		**6**
RKM	37	43	11	**91**
Vilbaste	343	383	90	**816**
SUM	**434**	**503**	**135**	**1072**

**Table 3:** Plants and their uses from folklore collections.

Family	Taxa	Local Name	Etic Disease Category	UR
Acoraceae	*Acorus calamus* L. ^L^	Jõekalmus ^S^, lesnagud ^S^, kalmus, tatersäla ^V^, kalmusejuur	Cardiovascular	1
			Culture-bound disease	1
			Digestive	7
			General	7
			Musculoskeletal	3
			Skin	1
			Urological	1
Amaranthaceae	*Beta vulgaris* L. ^C^	peet ^S^, verevä nakri ^S^	Eye	1
			Skin	4
Amaryllidaceae	*Allium cepa* L. ^C^	sibul, sippul ^S^, sipul ^V^	Cardiovascular	2
			Digestive	7
			Ear	1
			General	5
			Respiratory	5
			Skin	8
			Urological	1
	*Allium sativum* L. ^C^	kurslaga ^S^, kurslakk ^S^, küüslauk ^S^	Digestive	1
			General	1
			Respiratory	1
			Skin	1
Apiaceae	*Angelica sylvestris* L. ^L^	heinputk ^V^, pütsk ^V^	Digestive	2
	*Carum carvi* L. ^L^	köömned ^V^, küümned	Cardiovascular	1
			Digestive	5
			General	6
			Respiratory	2
Asparagaceae	*Convallaria majalis* L. ^L^	maikelluke ^S^	General	1
Asphodelaceae	*Aloe arborescens* Mill. ^C^	aalo, aaloe, aalus ^S^, aleo ^S^, pakso lill ^S^	Culture-bound disease	1
			General	6
			Respiratory	1
			Skin	7
Asteraceae	*Achillea millefolium* L. ^L^	raudrohi, verihein, verihain, valgõ lill ^S^	Culture-bound disease	2
			Digestive	11
			Female genital	2
			General	5
			Musculoskeletal	1
			Neurological	1
			Respiratory	6
			Skin	10
	*Antennaria dioica* (L.) Gaertn. ^L^	kassikäpad ^S^	Female genital	1
	*Arctium tomentosum* Mill. ^L^	takjas, takk ^S^	Blood	2
			Culture-bound disease	3
			Digestive	3
			General	3
			Respiratory	7
			Skin	11
			Urological	1
	*Artemisia absinthium* L. ^L^	koirohi, pänül ^S^, pälüm	Culture-bound disease	2
			Digestive	5
			General	3
			Musculoskeletal	1
			Skin	4
	*Carduus crispus* L. *or Cirsium vulgare* (Savi) Ten. ^L^	karuohtja ^V^	Urological	1
	*Gnaphalium uliginosum* L. ^L^	sammaspoolehain, sammaspoolehein, sammaspoolhain ^S^, sammaspoolikuhain ^S^, sammaspoolikuhein ^V^, sammaspoolõhain ^S^, soo-kassiurb ^V^	Skin	10
	*Matricaria chamomilla* L. ^C^	kamel ^V^, teekummel	General	6
			Neurological	4
			Respiratory	2
	*Matricaria discoidea* DC. ^L^	kaamel ^V^, kammel ^V^, kumelitee ^V^, kaamelihain ^V^, kummel, kummulid ^S^, lõhnav kummel, ubinhain, ubinhein, unõhain ^S^, upinhain, uppinhain ^S^	Culture-bound disease	2
			Digestive	9
			Ear	1
			Eye	3
			General	15
			Musculoskeletal	1
			Neurological	2
			Pregnancy, childbearing, etc.	1
			Respiratory	11
			Skin	6
	*Scorzoneroides autumnalis* (L.) Moench^L^*, Solidago virgaurea* L. ^L^ or with lower probability, many other taxa	arnikas, ärnika ^S^	Culture-bound disease	6
			Digestive	3
			General	2
			Neurological	1
			Respiratory	2
	*Solidago virgaurea* L. ^L^	ärnetsa ^V^	Musculoskeletal	1
	*Tanacetum vulgare* L. ^L^	kolladsõ lill ^S^, kollane lill ^S^, solknaheinad ^S^, solknarohi ^V^	Digestive	4
	*Taraxacum officinale* F.H. Wigg. ^L^	võilill	Digestive	1
			Skin	1
	*Tragopogon pratensis* L. ^L^	piimjuur ^V^	Digestive	1
	*Tripleurospermum inodorum* (L.) Sch.Bip. ^L^	kammel ^S^	Digestive	1
			Respiratory	1
	*Tussilago farfara* L. ^L^	ämmaleht ^V^	Skin	1
Betulaceae	*Alnus* spp. ^L^ (incl. *Alnus glutinosa* (L.) Gaertn. and *Alnus incana* (L.) Moench)	lepp, soolepp ^S^ imälepp ^S^ imälepp ^S^ valge lepp ^V^	Culture-bound disease	2
			Skin	4
			Digestive	1
	*Betula* spp. ^L^	kask, kõiv, kõo ^S^	Culture-bound disease	2
			Digestive	2
			General	5
			Musculoskeletal	7
			Respiratory	2
			Skin	8
Brassicaceae	*Armoracia rusticana* G. Gaertn., B. Mey. & Scherb. ^C^	maarjaritska ^V^, mädarõigas ^V^	Culture-bound disease	1
			Digestive	2
	*Brassica oleracea* var. *capitata f. Alba*^C^	kapsas ^S^	Eye	1
			Neurological	2
			Skin	1
	*Brassica rapa* L. ^C^	naar ^V^	General	1
	*Capsella bursa-pastoris* (L.) Medik. ^L^	hiirekõrv ^V^	Musculoskeletal	1
	*Sinapis alba* L. ^C^	sinep ^V^	Eye	1
Cannabaceae	*Cannabis sativa* L. ^C^	kanebi, kanep ^V^	Culture-bound disease	3
			General	1
Caprifoliaceae	*Valeriana officinalis* L. ^L^	balderjan ^V^, palderjaan ^V^, palderjan, paltõjan ^S^	Cardiovascular	3
			Culture-bound disease	7
			Digestive	17
			Female genital	1
			General	2
			Neurological	20
Crassulaceae	*Hylotelephium maximum* (L.) Holub ^L^	kidsihain, kidsihein ^V^, maapähkme ^V^	Culture-bound disease	7
			General	1
	*Sempervivum globiferum* L. ^N^	maasibul ^V^	Neurological	1
Cupressaceae	*Juniperus communis* L. ^L^	kadajas, kadakas, kadak ^V^, kattai	Blood	1
			Culture-bound disease	4
			Digestive	3
			General	19
			Musculoskeletal	1
			Neurological	2
			Respiratory	1
			Urological	8
	*Linum usitatissimum* L. ^C^	lina	General	3
			Skin	6
Cyperaceae	*Eriophorum vaginatum* L. ^L^	pikki hain ^V^	Skin	1
Droseraceae	*Drosera rotundifolia* L. ^L^	huulehain ^S^, huulhain ^S^, huulhein ^S^	Skin	5
Equisetaceae	*Equisetum arvense* L. ^L^	põldosi ^V^, tilkhain ^V^	Skin	1
	*Equisetum* spp. ^L^	osjad ^V^	Urological	1
Ericaceae	*Andromeda polifolia* L. ^L^	tsihknaõied ^S^	Respiratory	1
	*Arctostaphylos uva-ursi* (L.) Spreng. ^L^	leesikad, tsiamarja ^S^, lehike ^S^, tsiapalohka ^S^, tsiapalokka ^S^	Culture-bound disease	1
			General	1
			Respiratory	1
			Urological	2
	*Calluna vulgaris* (L.) Hull ^L^	kanarik ^S^, kanarpik ^S^, palokanarik ^S^	Digestive	1
			General	4
			Musculoskeletal	2
			Skin	3
	*Chimaphila umbellata* (L.) W.P.C.Barton ^L^	obijoinihain ^S^, obijoinilill ^S^, obijoon ^S^, opijon ^S^, obijoon ^S^, oobium, oopiumiheinad ^V^, opijann ^S^	Cardiovascular	2
			Culture-bound disease	3
			Digestive	4
			General	1
			Neurological	2
			Respiratory	4
	*Rhododendron tomentosum* Harmaja ^L^	sookael ^V^, sookanarbik ^S^, sookanarik ^S^, suukanarik ^S^ sootsähknad ^V^, soovitsked ^V^, tsihk ^S^, tsihkna ^S^	Digestive	2
			Respiratory	1
			General	4
			Musculoskeletal	2
			Skin	1
	*Vaccinium myrtillus* L. ^L^	mustikas, mustkas ^S^, mustigõ ^S^	Digestive	13
	*Vaccinium oxycoccos* L. ^L^	jõhvikad ^V^, kuremari ^S^, kuremarjad	Digestive	1
			General	2
			Neurological	1
			Skin	1
	*Vaccinium vitis-idaea* L. ^L^	palõhk ^S^, palohkna ^S^, palovka ^S^, palukas, pohl, pohlak ^V^	Culture-bound disease	1
			General	5
			Musculoskeletal	2
			Neurological	1
			Respiratory	5
			Urological	2
Fagaceae	*Quercus robur* L. ^L^	tamm, tammõ ^S^	Blood	1
			Culture-bound disease	2
			Digestive	4
			General	3
			Skin	5
Gentianaceae	*Gentiana cruciata* L. ^E^	vaivajarohi ^V^	Musculoskeletal	1
	*Gentiana* spp. ^E^	süäme alodsõ hain ^S^, südamealuse heinad ^S^	Cardiovascular	3
Grossulariaceae	*Ribes nigrum* L. ^C^	mustad hõrakad ^V^	General	1
Hypericaceae	*Hypericum* spp. ^L^	naistepuna ^V^	Digestive	1
			Female genital	1
Lamiaceae	*Mentha spicata* L. ^C^	rohemünt ^V^	Digestive	1
	*Mentha aquatica* L. ^L^	vesimünt ^S^	Neurological	1
	*Mentha* spp. ^C^	piparmünt, münt ^V^, aia-vehverments ^V^, pibarment ^S^, vehverloints ^S^, vehvermänts ^V^, vehverments	Neurological	4
			Culture-bound disease	1
			Digestive	7
			Female genital	1
			General	7
			Respiratory	5
	*Thymus serpyllum* L. ^L^	jaanihaina ^S^, jaanihein, kadedushein ^V^, kaetiserohi ^V^, üheksahaiguserohi ^V^, kolmekordne rohi ^V^, liivatee, üheksatõverohi ^S^, maarjahein, pühamaarjahaina ^S^	Digestive	2
			Eye	2
			General	12
			Musculoskeletal	3
			Neurological	1
			Pregnancy, childbearing, etc.	1
			Respiratory	11
	*Trifolium pratense* L. ^L^	ristikhein	General	1
			Musculoskeletal	2
			Respiratory	1
			Skin	1
	*Trifolium repens* L. ^L^	valge ristikhein	Female genital	1
			General	1
	*Trifolium* sp. ^L^	maarjaristikhein	General	1
Lauraceae	*Laurus nobilis* L ^P^	loorber ^V^	Blood	1
Lycopodiaceae	*Huperzia selago* (L.) Bernh. ex Schrank & Mart. ^N^	nõiakõld ^S^, nõiakollad ^V^	Cardiovascular	1
			Eye	1
	*Lycopodium clavatum* L. ^N^	karukollad ^V^, nõiakollad ^V^	Skin	1
Melanthiaceae	*Paris quadrifolia* L. ^L^	–	Digestive	1
Menyanthaceae	*Menyanthes trifoliata* L. ^L^	ubalehe ^V^, ubaleht ^V^	Digestive	1
			General	2
Oleaceae	*Fraxinus excelsior* L. ^L^	saar ^V^	Musculoskeletal	1
Orchidaceae	*Dactylorhiza maculata* (L.) Soó ^N^	jumalakäpp ^V^, juudakäpp ^V^	Digestive	1
Papaveraceae	*Chelidonium majus* L. ^L^	vererohi ^V^	Urological	1
	*Corydalis solida* (L.) Clairv ^L^.	vaivaja haina ^S^	Culture-bound disease	2
	*Fumaria officinalis* L. ^L^	juuksehain ^V^	Skin	1
	*Papaver somniferum* L. ^C^	magun ^S^, makunna ^S^	Digestive	1
			Neurological	1
Pinaceae	*Picea abies* (L.) H.Karst. ^L^	kuus, kuusk	Digestive	1
			General	10
			Musculoskeletal	4
			Respiratory	2
			Skin	15
	*Pinus sylvestris* L. ^L^	mänd, pettai ^V^,petäi ^S^, pedäjäs ^S^	Digestive	2
			General	27
			Musculoskeletal	8
			Respiratory	11
			Skin	9
Piperaceae	*Piper nigrum* L. ^P^	pipar	Digestive	2
			Respiratory	1
Plantaginaceae	*Plantago major* L. ^L^	paiselehe, paiseleht, umbleht ^V^, ummelehe ^S^, teeleht ^V^	Culture-bound disease	1
			Eye	1
			General	2
			Musculoskeletal	1
			Skin	24
Poaceae	*Avena sativa* L. ^C^	kaar, kaer	Culture-bound disease	4
			Digestive	4
			General	5
	*Hordeum vulgare* L. ^C^	kesvad ^V^, oder ^V^	Skin	3
	*Secale cereale* L. ^C^	rüä ^S^, rüga ^V^, rukis	Digestive	2
			Musculoskeletal	1
			Skin	5
Polygonaceae	*Persicaria amphibia* (L.) Delarbre or *Glyceria maxima* (Hartm.) Holmb. ^L^	läsnäk ^S^, lesnak ^S^, lesnäk ^S^	General	3
			Respiratory	2
	*Polygala amarella* Crantz ^L^	vahulill ^S^	Skin	1
	*Polygonum arenastrum* Boreau ^L^	morohain ^S^, niseldushain ^V^, niseldushein ^V^, nisõldushaina ^V^	Musculoskeletal	3
			Skin	1
	*Polygonum aviculare* L. ^L^	–	Musculoskeletal	1
	*Rumex crispus* L. ^L^	kärnhain ^S^ hobuhain ^S^	Skin	2
			Respiratory	1
	*Rumex hydrolapathum* Huds. ^L^	kärnahain ^S^	Skin	2
Polypodiaceae	*Dryopteris filix-mas* (L.) Schott ^L^ or *Pteridium aquilinum* (L.) Kuhn ^L^; (Dennstaedtiaceae)	maarjasõnajalg ^V^, sõnajalg ^V^	Culture-bound disease	2
			Digestive	7
			Musculoskeletal	2
			Neurological	1
Ranunculaceae	*Anemone nemorosa* L. ^L^	haragheinad ^S^, haraklilled ^S^	Digestive	2
Rhamnaceae	*Frangula alnus* Mill. ^L^	kisõpuu ^S^, kitsepuu ^S^, kitsetoome ^V^, kitseuibo, vohopaadsa, soemära ^S^, paakspuu	Digestive	8
Rosaceae	*Alchemilla vulgaris* L. ^L^	kortsleht ^V^	Digestive	1
	*Potentilla argentea* L. ^L^	verehain ^S^	Skin	1
	*Filipendula ulmaria* (L.) Maxim. ^L^	angervaks ^V^	Pregnancy, childbearing, etc.	1
			Skin	1
	*Fragaria vesca* L. ^L^	maasikas	General	3
			Respiratory	4
	*Malus domestica* (Suckow) Borkh. ^C^	õunapuu ^S^, uibu ^S^, uip ^S^, uipoh ^S^	Culture-bound disease	1
			General	3
			Respiratory	2
	*Potentilla erecta* (L.) Raeusch. ^L^	kalkanajuured ^S^, kalgan ^V^, maramaar ^S^, maran, nabahain ^V^, tedremadar ^V^, tedremaran, tedremarja juured ^S^	Blood	1
			Culture-bound disease	6
			Digestive	14
			General	1
	*Prunus cerasus* L. ^C^	kirss, vislapuu ^S^	Digestive	1
			Respiratory	2
	*Prunus padus* L. ^L^	toome, toomingas	Cardiovascular	1
			Digestive	14
			General	7
			Neurological	3
			Respiratory	2
			Skin	3
	*Pyrus communis* L. ^C^	pruusa ^S^	Culture-bound disease	1
	*Rubus chamaemorus* L. ^L^	murakad ^S^	Musculoskeletal	2
	*Rubus idaeus* L. ^L^	vabarna	Digestive	1
			General	10
			Respiratory	7
	*Rubus polonicus* Weston ^L^	mustad vabarnad	General	1
	*Sorbus aucuparia* L. ^L^	pihlakas, pihl ^S^, pihlapuu ^S^	Culture-bound disease	4
			Digestive	4
			General	7
			Respiratory	3
			Skin	2
Rubiaceae	*Coffea* sp. ^P^	kohv ^V^	Culture-bound disease	1
			Respiratory	1
	*Galium boreale* L. ^L^	niseldushain, nikastushein	Musculoskeletal	1
Salicaceae	*Populus tremula* L. ^L^	haab	General	2
	*Salix* spp. ^L^	pai ^S^, paju	Digestive	1
			General	1
			Respiratory	2
			Skin	8
Sapindaceae	*Acer platanoides* L.^L^	vaher ^V^	General	1
Solanaceae	*Capsicum annuum* L. (Longum Group) ^C^	kõdrapipar ^S^, pipar söögipipõr ^S^, türgi pipar ^V^, verevä pipar ^S^ verikõder, veripipõr ^S^	Digestive	22
			General	1
			Musculoskeletal	2
			Respiratory	6
	*Hyoscyamus niger* L. ^N^	hambahain ^S^	Digestive	2
	*Nicotiana rustica* L. ^C^	tubaguhain ^S^, tubak ^S^, tubakas, tubakulehe ^V^	Culture-bound disease	4
			Digestive	2
			Respiratory	1
			Skin	2
	*Solanum dulcamara* L. ^L^	maavitsad ^V^, päris maavits	Musculoskeletal	6
			Skin	6
	*Solanum tuberosum* L. ^C^	kartohvel ^S^, kartokas ^S^, kartul	Culture-bound disease	1
			Digestive	2
			Musculoskeletal	1
			Skin	2
Thymelaeaceae	*Daphne mezereum* L. ^L^	küüvits ^V^	Digestive	3
Tiliaceae	*Tilia cordata* Mill. ^L^	lõhmus, pahka ^V^, pähn ^V^, pärn, pähnäpuu ^V^, pärnapuu ^V^	Blood	2
			Digestive	2
			General	20
			Respiratory	10
			Skin	4
			Urological	1
Urticaceae	*Urtica dioica* L. ^L^	nõges	General	2
			Musculoskeletal	4
			Respiratory	2
			Skin	2
	*Urtica urens* L. ^N^	raudnõges ^S^	General	2
			Skin	2
Viburnaceae	*Viburnum opulus* L. ^L^	lodjapuu	Digestive	3
			General	1
			Neurological	1
			Respiratory	2
	*Adoxa moschatellina* L. ^L^	mättahain ^S^, mättalill ^S^	Respiratory	1
			Cardiovascular	1
*Unidentified taxon*		härjapää ^V^, verihein ^V^	Respiratory	1
		kandrohi ^S^	Digestive	2
			Female genital	1
		karamarjad ^S^	Urological	1
		kärnõ rohi ^S^	Skin	1
		lepakukud ^S^	General	1
		luuvaluheinad ^V^	Musculoskeletal	1
		nätselmehein ^V^	General	2
			Musculoskeletal	1
		palohain ^S^	Culture-bound disease	1
		pinipussuhain ^S^	General	1
		punatse lill ^V^	Female genital	1
		tõrvaleht ^V^	General	1
		tõrvaõied ^S^	Digestive	1
		valge kassikäpp ^V^	Female genital	1
		valge lill ^V^	Female genital	1
		valge lill	Respiratory	2
		valgõ lill	digestive	2
		no name ^S^	Skin	1

Unless recorded in both: ^S^—Local plant names recorded in Setomaa, ^V^—local plant names recorded in Võromaa; ^C^—Cultivated, ^L^—Least Concern, ^N^—Near Threatened, ^E—^Endangered, ^P^—does not grow in Estonia. Extinction risk statuses were taken from the Estonian red list, as specified in the database at https://elurikkus.ee/ (accessed on 28 September 2022).

**Table 4:** Folklore collections and the number of pre-selected text segments from every collection. Source: https://www.folklore.ee/era/leidmine/index.html (accessed on 10 July 2022).

Abbreviation	Full Name of the Collection	Years of Collection	No. of Pages of Full Collection	No. of Texts in Setomaa	No. of Texts in Räpina	No. of Texts in Vastseliina
H	Folklore collection of J. Hurt	1860–1906	114,696	40	17	27
E	Folklore collection of M. J. Eisen	1880–1934	90,100	1	5	2
ERM	Folklore collection of Estonian National Museum	1915–1925	9398			4
ERA	Folklore collection of Estonian Folklore Archives	1927–1944	265,098	39	28	1
Vilbaste	Folklore collection of G. Vilbaste	1907–1966	20,327	520	309	196
KKI	Institute of Language and Literature folklore collection	1941–1984	35,679	6		
RKM	Folklore collection of Folklore Department of Estonian Literary Museum	1945–1996	447,231	35	37	7
		**SUM of records**	**982,529**	**631**	**400**	**237**

## Data Availability

Data are available in the Supplementary file.

## Supplementary Materials

The following supporting information can be downloaded at: https://www.mdpi.com/article/10.3390/plants11202698/s1, Table S1: Systematized texts.
